# Treatment Strategy in Atrial Tachycardia Originating From the Atrial Appendage

**DOI:** 10.3389/fphys.2022.902513

**Published:** 2022-06-24

**Authors:** Xie Hai-Yang, Feng Zi-Cong, Guo Xiao-Gang, Sun Qi, Yang Jian-Du, Ma Jian

**Affiliations:** ^1^ State Key Laboratory of Cardiovascular Disease, Fuwai Hospital, National Center for Cardiovascular Diseases, Chinese Academy of Medical Sciences and Peking Union Medical College, Beijing, China; ^2^ Department of Cardiology, Sun Yat-Sen Memorial Hospital, Sun Yat-Sen University, Guangzhou, China; ^3^ Department of Cardiac Surgery of the First Affiliated Hospital of Sun Yat-Sen University, Guangzhou, China

**Keywords:** catheter ablation, atrial appendage, atrial tachycardia, atrial appendectomy, ivabradine

## Abstract

**Background:** Atrial appendage tachycardia (AAT) originating from the atrial appendage (AA) is extremely difficult to eliminate using radiofrequency catheter ablation (RFCA). The optimal management strategy for AAT refractory to RFCA remains unclear.

**Objective:** This study aims to investigate the long-term result of ablative therapy and the optimal alternative management for AAT refractory to RFCA.

**Methods:** A total of 51 patients with AAT originating from the AA undergoing RFCA were recruited. Video-assisted atrial appendectomy and oral ivabradine were performed on those with AATs refractory to RFCA, and this study aimed to evaluate their safety and long-term efficacy.

**Results:** We included 51 patients (51/586, 8.7%) with AATs confirmed by activation mapping and contrast venography. Among them, there were 28 (54.9%) AATs originating from the distal AA. In total, 14 (27.4%) AATs were refractory to RFCA, including 13 originating from the distal AA and one arising from the proximal AA. Ten of 11 (90.9%) AATs originating from the distal AA were eliminated after an atrial appendectomy, and the other three AATs were suppressed using oral ivabradine. Origins from the distal AA refractory to RFCA and early age of AAT onset ≤26.5 years indicated the need for atrial appendectomy. No major complications occurred, and nine patients with tachycardia-induced cardiomyopathy fully recovered. Long-term success was achieved in 98.0% of patients with multiple treatment managements.

**Conclusion:** AATs originating from the distal AA were more refractory to RFCA. RFCA was the cornerstone of AAT catheter ablation. Video-assisted thoracoscopic atrial appendectomy was an effective strategy for those origins at the distal AA and the age of AAT onset ≤26.5 years. Ivabradine represents a promising treatment for AAT temporarily in pediatric and young adult patients.

## Introduction

Focal atrial tachycardias from the right atrial appendage (RAA) are uncommon, with a prevalence of 3.8–8% (Roberts-Thomson et al., 2007; Freixa et al., 2008), and those from the left atrial appendage (LAA) occur with a prevalence of 2.1% (Yang et al., 2012). Their electrophysiological features have been well described previously (Roberts-Thomson et al., 2007; Yamada et al., 2007; Freixa et al., 2008; Yang et al., 2012). Although rare, atrial appendage tachycardia (AAT) tends to be incessant and increases the risk of tachycardia-induced cardiomyopathy (TCM) with a fast ventricular response. Consequently, aggressive management is needed. Considering that the effectiveness of antiarrhythmic drugs for this set of tachycardias is limited, radiofrequency catheter ablation (RFCA) has been considered a curative treatment thus far and has yielded a satisfying outcome. However, successful catheter ablation in this anatomic region can be difficult in some cases. Worst of all, mapping and radiofrequency ablation within the thin-walled atrial appendage may pose the risks of perforation and cardiac tamponade. Apart from surgical alternatives, cryoballoon ablation to isolate the AA orifice or focal cryoablation could be applied endocardially (Roshan et al., 2015; Yorgun et al., 2017). Furthermore, epicardial RF ablation can be applied as an alternative if the area cannot be reached from the endocardial site. To date, there have been only sporadic systematic reports evaluating the management of atrial appendage tachycardia refractory to RFCA. Therefore, the primary goal of this study was to evaluate the long-term results of ablative therapy for AAT and to investigate the alternative management and long-term outcomes of AAT refractory to RFCA.

## Methods

### Study Population

From April 2004 to January 2020, a total of 51 patients with AAT (8.7%) originating from the atrial appendage were recruited from 586 consecutive patients undergoing RFCA for focal atrial tachycardia. Clinical evaluation with an electrocardiogram (ECG), transthoracic echocardiography, and lab tests was performed before the procedure. All patients provided written informed consent before the procedure. This study was approved by the Institutional Review Board of Fuwai Hospital.

### Electrophysiological Study

**FIGURE 1:**
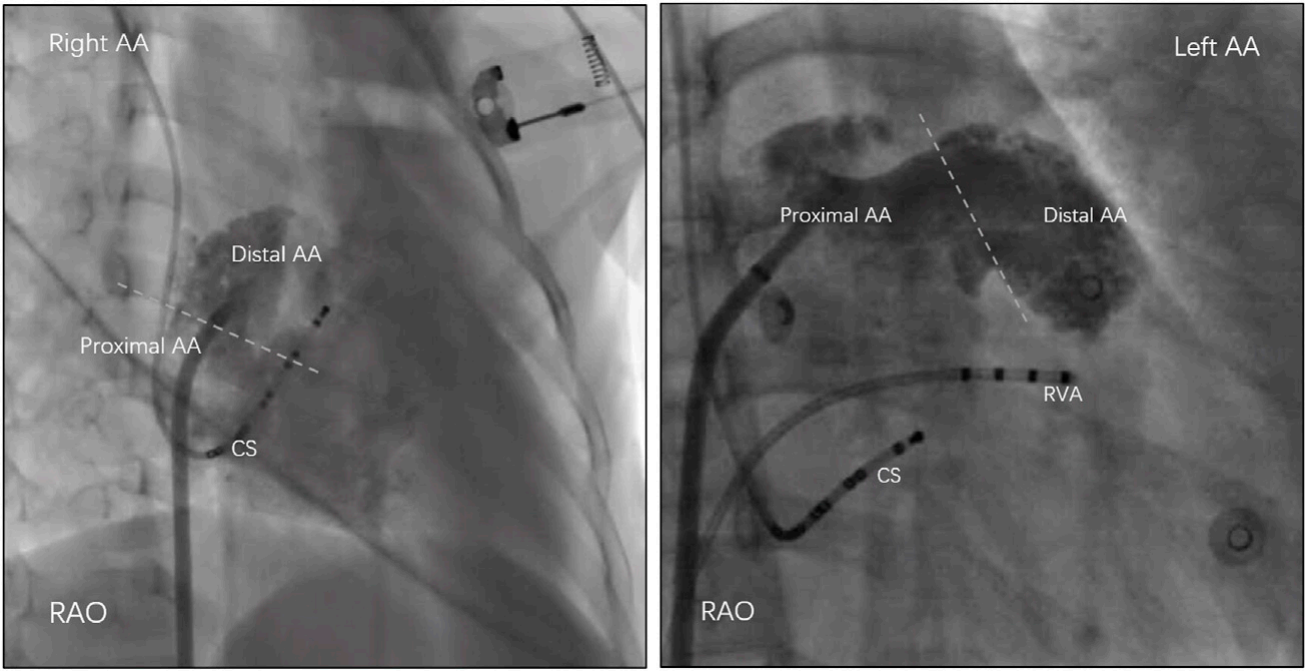
The atrial appendage (AA) was arbitrarily divided into two sections by contrast venography: proximal AA and distal AA. In detail, the proximal portion had a smooth endocardial contour, while the distal portion that was highly trabeculated had an uneven contour. RAO = right anterior oblique; CS = coronary sinus; RVA = right ventricular apex.

All antiarrhythmic drugs were withdrawn at least five half-lives before the procedure. For each patient, three catheters were introduced into the right ventricular apex, at the His bundle electrogram region, and into the coronary sinus. Data were recorded simultaneously by a digital multichannel system (LabSystem PRO, Bard Electrophysiology, Lowell, MA, United States). Bipolar signals were filtered at 30–500 Hz, and unipolar signals were filtered at 0.05–500 Hz. Standard electrophysiological criteria were used to diagnose AT (Lesh et al., 1996). If AT was not present at the beginning of the procedure, programmed atrial pacing and burst atrial pacing, with intravenous isoproterenol if necessary, were applied to induce clinical AT. Electroanatomical mapping was performed using the CARTO system (Biosense Webster Inc., Diamond Bar, CA, United States) to facilitate mapping and assessment of the anatomic location. Point-by-point high-density mapping was performed in the area with early activation in the atrium. The earliest atrial activation site was identified by measuring the activation time relative to the onset of the P wave, and the location was confirmed by anterograde venography. Before detailed mapping in the appendage, contrast venography (15–20 ml contrast agent per injection) with a steerable sheath was performed to maximize visualization of the appendage under a suitable view. Based on this view, contrast venography (containing 50% contrast agent and saline, 2–3 ml) via the catheter tip was performed after identifying the earliest activation site but before ablation. As described previously (Guo et al., 2014), the AA was arbitrarily divided into two sections by contrast venography: proximal AA and distal AA. In detail, the proximal portion had a smooth endocardial contour, while the distal portion that was highly trabeculated had an uneven contour (Figure 1).

### Radiofrequency Catheter Ablation

An irrigated tip catheter was applied to deliver radiofrequency energy with a temperature limited to 43–45°C and saline flow of 17–20 ml/min. At the distal AA, radiofrequency energy started at 20 W and gradually increased up to 30 W, while at the proximal portion, a power of 30–35 W was delivered. Radiofrequency ablation was maintained for 90 s when effective. However, if tachycardia did not terminate within 15 s, the catheter would be dislodged. An ablation attempt was regarded as a feasible endpoint by the absence of tachycardia 60 min after ablation despite an infusion of isoproterenol (up to 6 μg/min) and aggressive burst atrial pacing.

### Video-Assisted Thoracoscopic Atrial Appendectomy

For AATs refractory to RFCA, if foci were observed at the distal AA, video-assisted thoracoscopic (VAT) atrial appendectomy was suggested. All antiarrhythmic drugs were withheld for at least five half-lives before surgery. Minimally invasive VAT atrial appendectomy was performed under general anesthesia. The atrial appendage was clamped and excised from the base using an endoscopic linear staple (Endo GIA, United States Surgical Corp, Norwalk, VA). Immediate termination of the AAT after excising from the base was regarded as an acute success.

### Ivabradine Therapy

For patients with RFCA failure who refused to undergo atrial appendectomy, oral ivabradine was administered at a dosage of 5 mg twice a day for adults, while a weight-adjusted dose (0.14 mg/kg/day) divided into two doses was used in pediatric patients ≤50 kg (Banavalikar et al., 2019; Michel et al., 2020). The ivabradine response was defined as the termination of AAT with the restoration of sinus rhythm within 12 h of initiating ivabradine.

### Follow-Up

After the procedure or taking ivabradine, continuous telemetry monitoring was performed the day after treatment in all patients. Transthoracic echocardiography and Holter monitoring were evaluated in the outpatient clinics at 1, 3, 6, and 12 months and yearly thereafter.

### Statistical Analysis

Continuous variables were presented as the mean ± SD, and were assessed using Student’s t-test or the Mann–Whitney test as appropriate. Categorical variables were presented as counts (percentage) and analyzed using the chi-square test or Fisher’s exact test. Sensitivity and specificity for each variable were determined using the receiver operator characteristics (ROC) curve. All clinically relevant variables were included in the multivariable logistic regression to identify the variables independently associated with the presence of a second loop. A value of *p* < 0.05 was considered significant. SPSS version 19.0 (SPSS, Inc., Chicago, IL, United States) was used for statistical analysis.

## Results

### Patient Characteristics

The study population consisted of 51 patients (22 men; mean age, 29 ± 17 years). Table 1 summarizes the baseline characteristics of all patients. Nine patients (17.6%, 27.5 ± 10.5 years old) had complications due to TCM (Table 2). There was no significant difference in baseline characteristics compared with patients without TCM. Eighteen (35.3%) patients had previous failed ablation attempts or experienced recurrence, and one had a history of an atrial appendectomy at other centers.

**TABLE 1:** Baseline characteristics.

	AAp (*N* = 23)	AAd (*N* = 28)	*p* value
Age	37.8 ± 19.4	20.6 ± 9.3	<0.001
Age of AAT onset	34.3 ± 17.8	15.8 ± 7.9	<0.001
Gender: female	11 (47.8%)	19 (50.0%)	0.148
TCM	4 (17.4%)	5 (17.9%)	0.728
AADs	1.5 ± 0.5	1.6 ± 0.7	0.908
Focus in LAA	11 (47.8%)	18 (64.3%)	0.238
No. of RFCA attempts	1.3 ± 0.5	1.7 ± 0.8	0.05

Values are presented as the mean ± SD and as n (%).

AAT = atrial appendage tachycardia; LAA, = left atrial appendage; AAD = antiarrhythmic drug; TCM = tachycardia-induced cardiomyopathy; RFCA = radiofrequency catheter ablation.

**TABLE 2:** Clinical and electrophysiological characteristics in patients with TCM.

Case	Age (year)	Age of onset (year)	History (year)	Gender	Focus	Focus in distal	TCL (ms)	Treatment	LVEF before treatment (%)	LVEF after treatment (6 months, %)
1	28	27	1	Female	LAA	No	470	RFCA	35	62
2	46	42	4	Female	RAA	Yes	476	RFCA	19	55
3	14	13	1	Male	LAA	Yes	306	RFCA	22	60
4	37	35	2	Female	LAA	No	440	RFCA	32	58
5	31	29	2	Male	LAA	No	310	RFCA	42	65
6	25	7	18	Female	LAA	Yes	390	RFCA	40	62
7	13	11	2	Male	RAA	No	450	RFCA	44	63
8	32	25	7	Female	LAA	Yes	360	VAT	38	60
9	22	11	11	Female	LAA	Yes	340	VAT	43	63
*p* value^a^	0.326	0.459	0.936	0.565	0.582	0.797	0.625	—	—	—

a Comparison with patients without TCM.

LVEF = left ventricular ejection fraction; RFCA = radiofrequency catheter ablation; AA = atrial appendage; VAT atrial appendectomy = video-assisted atrial appendectomy; TCM = tachycardia-induced cardiomyopathy; TCL = tachycardia length.

### Radiofrequency Catheter Ablation Outcomes

**FIGURE 2:**
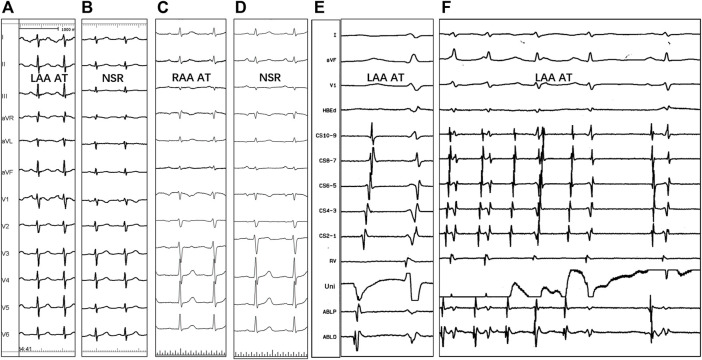
P-wave morphology of the left **(A,B)** and right **(C,D)** atrial appendage tachycardias, the electrogram of successful ablation site **(E)**, and tachycardia termination with RF application **(F)**. NSR = normal sinus rhythm; Uni = unipolar electrogram; ABLP and ABLD = bipolar electrogram; Other abbreviations as previously.

Spontaneous tachycardia was present in 48/51 (94.1%) patients, while clinical tachycardia was induced in the lab in the remaining three patients. Catheter mapping revealed 29 AATs originating from LAA (56.9%) and the other 22 AATs from RAA (43.1%). The P-wave morphology of the left AAT in all patients showed a negative P wave in leads I and aVL (Figure 2). Additionally, the P wave was positive in leads II, III, and aVF and broadly positive in lead V1 in all patients. However, the ECG of right AAT was only characteristic in lead V1, which showed negative P waves (21 of 22 patients, 95.5%). P waves in lead I were positive (19 of 22 patients, 86.4%) and positive in inferior leads (18 of 22 patients, 81.8%).

Twenty-eight AATs (54.9%) were found at the earliest activation site located at the distal AA, while the other 23 (45.1%) were at the proximal AA. There was no low-voltage zone (<0.5 mV) found in and around the appendages of these patients. Catheter ablation achieved acute success in 44 AATs (86.3%, 100% for proximal AA but 75.0% for distal AA), and tachycardias could not be eliminated in the remaining seven patients even if repeat mapping and ablation were performed. The mean RFCA application times (90 s each time) per patient in the RAA and LAA were 3.7 ± 1.8 (range: 1–10) and 3.9 ± 2.3 (range: 1–9), respectively (*p* = 0.731). During a mean follow-up period of 5.1 ± 3.5 years, nine patients experienced recurrences (1–208 days, median: 2 days), including five patients at distal LAA, three patients at distal RAA, and one patient at proximal RAA near the sinus node. The mean RFCA application time in this group was greater than that in the group without recurrence (4.6 ± 1.4 vs 2.9 ± 1.0, *p* < 0.05). Four patients received a redo procedure, and half of them were free from AATs. In addition, one patient received a third RFCA but still experienced recurrence during follow-up. Among the 14 AATs refractory to RFCA, the origin was located at the distal AA in thirteen patients (92.9%) and the proximal AA in one patient (7.1%).

### Video-Assisted Atrial Appendectomy Outcomes

Of 14 patients with RFCA failure, 11 who were identified by contrast venography and electroanatomical mapping to have the origin at the distal portion of LAA or RAA agreed to undergo VAT atrial appendectomy. All AATs were immediately terminated when the AA was excised at the level of clamping during the procedure. During a mean follow-up period of 4.1 ± 3.0 years, only one patient had recurrence on the day after surgery. No complications occurred in any patient.

**FIGURE 3:**
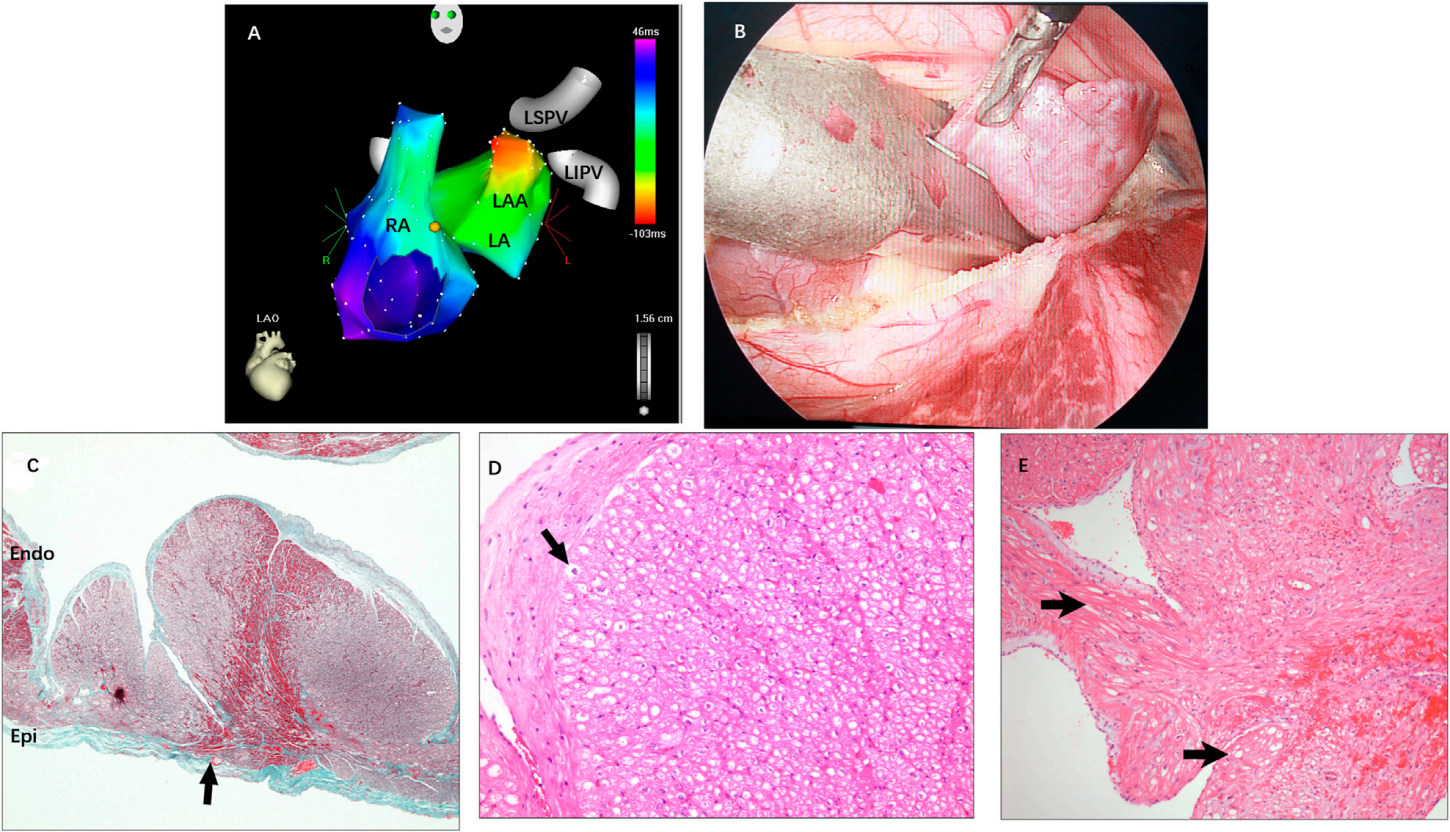
**(A)** Activation mapping of AAT originating from the distal LAA. **(B)** The LAA was clamped and excised from the base using an endoscopic linear staple. **(C)** Pathohistology of excised LAA: A transmural lesion was noted with granulation and fibrosis next to hemorrhage (arrow). **(D)** Mild vacuolar degeneration of cardiomyocytes in the apex of the LAA (arrow) and absence of sinoatrial node-like tissue. **(E)** The area in the vicinity of the lesion was interspersed with abundant nuclei-absent cells that clustered to form a kidney-shaped figuration (arrow). RA = right atrium; LA = left atrium; LSPV and LIPV = left superior/inferior pulmonary vein; Other abbreviations as previously.

Histopathological examination revealed nontransmural or transmural ablation cicatrices on the endocardial side in all specimens. In addition, mild vacuolar degeneration of cardiomyocytes in the apex of the LAA was seen in most of the specimens, but sinoatrial node-like tissue was not found in any specimens. In one specimen, the area in the vicinity of the lesion was interspersed with abundant nuclei-absent cells that clustered to form a kidney-shaped figuration, which corresponded to the automaticity cells described by de Barker et al. (1994) (Figure 3).

### Ivabradine Therapy Outcomes

For four patients with failure or refusal to undergo RFCA and atrial appendectomy, three patients (one with origin at the proximal AA and two in the distal AA) agreed to be treated with ivabradine. There was a termination of AAT with the restoration of sinus rhythm in all patients after the first dose of ivabradine. Ivabradine was well tolerated over a mean follow-up of 1.7 ± 0.6 years without any major complications or recurrence of AATs in any patient.

### Characteristics of Atrial Appendage Tachycardia

**FIGURE 4:**
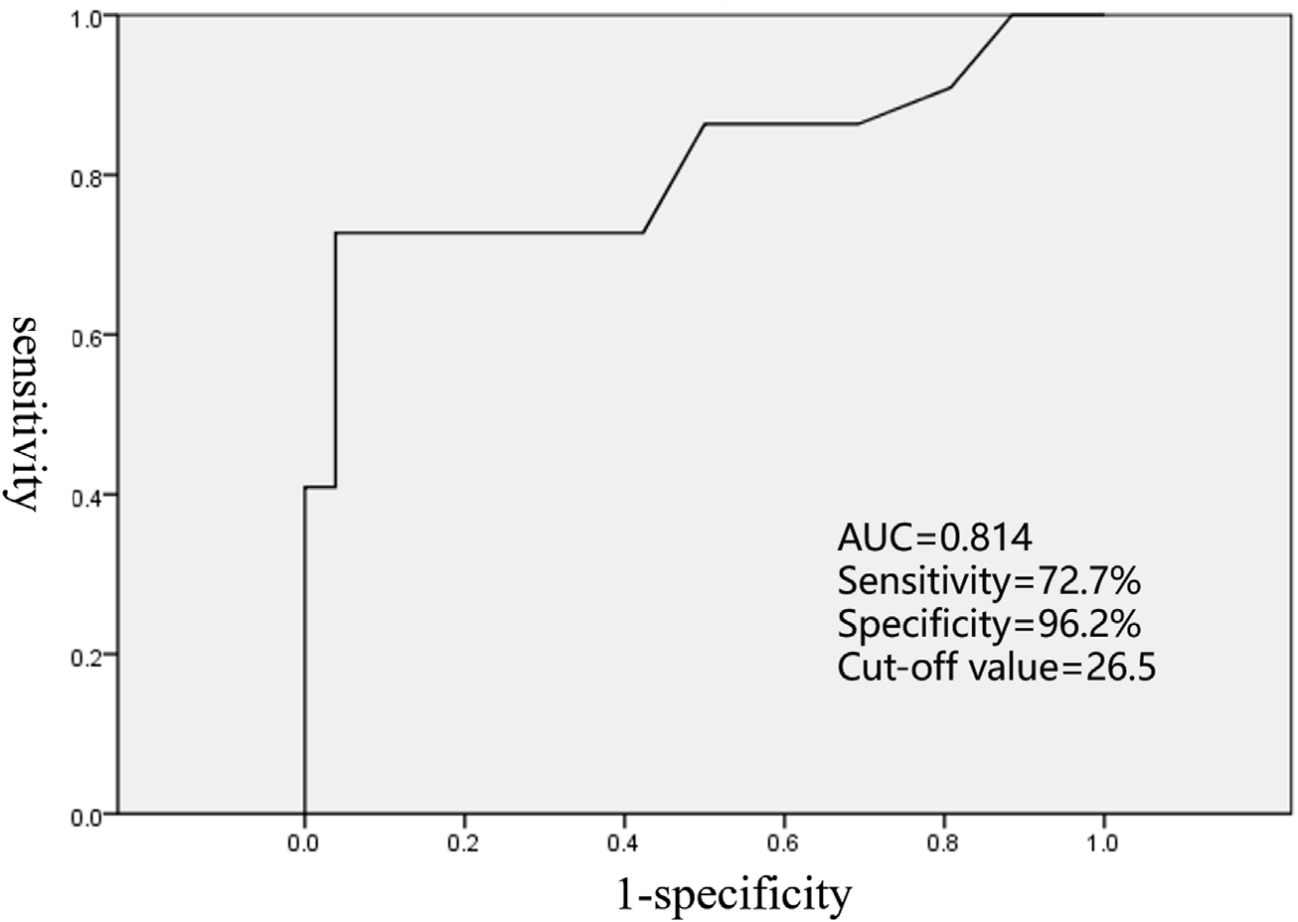
ROC curve analyzing the age of AAT onset to predict the origin location.

In the current study, there was no significant difference in the location of AAT origin between LAA and RAA (*p* = 0.238), but AAT originating from the distal AA mostly presented at a young age (*p* < 0.001). The ROC curve showed the cutoff value, and age of AAT onset ≤26.5 years was able to predict that the origin was at the distal AA (sensitivity of 72.7% and specificity of 96.2%, area under the receiver operating characteristic curve of 0.814, Figure 4). AAT arising at the distal AA tended to receive second or more ablation procedures (*p* = 0.05). In addition, the early age of AAT onset (*p* < 0.024) and the origin at the distal AA (*p* = 0.001) were predictors of AAT refractory to RFCA.

### Complications

In this series, there were no complications, including cardiac tamponade, atrioventricular block, vascular access complications needing surgical treatment, and periprocedural infection, bleeding, or thromboembolism. However, one patient developed a right femoral arteriovenous fistula that resolved spontaneously during the follow-up.

## Discussion

### Major Findings

To our knowledge, this is the largest series to validate the efficacy and safety of multiple alternative strategies in treating AAT with a very long follow-up period, including RFCA, VAT atrial appendectomy, and oral ivabradine. Origins at the distal portion of the AAT were mostly present among the young and easily recurrent patients treated by RFCA. When applying VAT atrial appendectomy, the origin of AAT should only arise at the distal AA. However, with late-onset (age >26.5 years) AAT, atrial appendectomy should be used with caution. Oral ivabradine was still effective and could be an alternative therapy for suppressing AAT that is refractory to the procedure. TCM resolved after treatment, and the average time was 3.6 ± 2.0 months.

### Treatment Strategy in Atrial Tachycardia Originating From the Atrial Appendage

Catheter mapping and ablation were the first-line diagnosis and therapy to eliminate AAT. Several systematic reports have demonstrated that the ablation results of AAT yielded a high rate of success (Yamada et al., 2007; Yun-Long et al., 2007; Freixa et al., 2008; Yang et al., 2012). However, the RFCA outcomes in some reports were still unfavorable. A considerable rate of recurrence or failure ranged from 10 to 28% (Roberts-Thomson et al., 2007; Yun-Long et al., 2007; Guo et al., 2014). Most of the AATs refractory to RFCA originated from distal AA. In the present study, 18/51 (35.3%) patients had undergone failed ablation attempts before, and 14 (77.8%) AATs arose from the distal AA. A total of 86.3% acute success rate was achieved by RFCA in our center, but nine experienced recurrence, including eight AATs originating from the distal AA and one from the proximal AA close to the sinus node. Consistent with previous studies, a high acute success rate and low recurrence was achieved among the origin at the proximal AA, but as for the origin at the distal AA, RFCA was still a challenge, even with multiple and diligent mapping and ablation. This could be explained by the unique and delicate nature of AA.

Usually, AA varies significantly in shape, size, and orientation to adjacent cardiac structures (Beigel et al., 2014). Compared with the proximal AA, distal AA, complicated with a multilobulated morphology, thin wall, and relatively low blood flow (Hillock et al., 2006; Yen Ho et al., 1999), often restricts catheter mobility and precisely delivers effective energy to the desired point. In addition, AA is a highly mobile structure that limits catheter maneuverability and consistent contact during ablation, even with a steerable sheath. The risk of complications, such as perforation from catheter manipulation or rupture from ablation steam pop, may thus increase with inadvertent manipulation and multiple procedures. As such, gentle manipulation and experienced hands are required to reach the tip. Otherwise, alternative methods are urgently needed to avoid such challenges. To overcome this problem, Chun et al. (2009) employed cryoballoon ablation and successfully isolated the RAA. Andreas et al. reported a high incidence of LAA thrombus formation and stroke after atrial appendage isolation. Whereas Yorgun et al. demonstrated no increase in thromboembolic complications (Rillig et al., 2016; Yorgun et al., 2017), the impairment in AA mechanical functions closely related to thromboembolic events (Goldman et al., 1999) was still seen in some patients. In addition, the efficacy of cryoballoon therapy is highly dependent on suitable appendage anatomy, which may not be suitable for everyone. As an alternative, Roshan et al. (2015) described the use of a cryocatheter to improve contact and increase the probability of approximating the focal origin of the tachycardia when conventional RF within the RA appendage proved unsuccessful. Phillips et al. (2008) applied a percutaneous pericardial approach that successfully eliminated AAT. Although effective, this invasive epicardial access may accompany the potential of various complications ranging between 5 and 10% due to patient-specific risk factors (Aryana et al., 2020).

Until now, several studies have demonstrated that VAT atrial appendectomy could safely and effectively eliminate AAT (Yamada et al., 2006; McGarvey et al., 2008; Furushima et al., 2009; Guo et al., 2014; Torok et al., 2014). Consistent with previous reports, atrial appendectomy was considered a feasible strategy to manage AAT refractory to RFCA in the present study. A total of 90.9% of patients were free from AATs without any antiarrhythmic drugs, and no extra complications occurred in any of the patients. Atrial appendectomy provided electrophysiologists with a method to manage AAT refractory to RFCA by avoiding worthless ablation. The P-wave morphology was unable to distinguish the AAT originating from the distal AA or proximal AA (Yang et al., 2012). In addition, the AA was clamped and excised from the base in the atrial appendectomy, but the proximal AA was not totally included. Thus, precise catheter mapping is required before resorting to atrial appendectomy. In the present study, the age of AAT onset was confirmed to be associated with the origin location. A cutoff age of AAT onset ≤26.5 years was able to predict the origin from the distal AA. The age of AAT onset in all successful surgery cases was under 26.5 years, except for the only one unsuccessful case among those 11 patients. Therefore, when deciding to resort to atrial appendectomy, the age of AAT onset might also be taken seriously into consideration. Late-onset AAT should be used with caution for atrial appendectomy.

The atrial appendage morphology of the patient (female, 52, suffering from tachycardia at the age of 47) who experienced recurrence after appendectomy presented a triangle shape without a long lower lobe. Electroanatomical activation mapping and contrast venography showed that the earliest activation was located at the middle-distal section of the RAA. Unfortunately, this patient was not willing to accept a second ablation. Therefore, we could not remap the focus. Several reasons might cause the recurrence of tachycardia. First, AA is a highly mobile structure and easily distorted when contracting and dilating or by catheter stress, and the focus might not be exactly located where we mapped it. Second, AA in theory was clamped and excised from the base when appendectomy was performed. However, it is possible to miss a residual root when an appendectomy is performed. Thus, if a residual root was left, high-frequency electrosurgical equipment could be considered to manage the remnant to reduce the possibility of recurrence.

AAT usually resists antiarrhythmic agents. In a study led by Bohora et al. (2011), one patient with incessant AAT was partially suppressed by ivabradine. Banavalikar et al. (2019) described six cases with AATs that were positive for ivabradine. In the present study, AATs originating from distal AA or proximal AA were all switched to sinus rhythm after the first dose of ivabradine. Ivabradine was well tolerated during the follow-up with no complications. Corresponding to other published reports, we have found it to be a safe agent with rapid response in treating AAT. Generally, ivabradine lowers heart rate by selectively inhibiting If channels, which are located at the sinoatrial node, in a concentration-dependent manner without affecting any other cardiac ionic channels (including calcium or potassium) (Koruth et al., 2017). First, we speculated whether sinoatrial node-like tissues existed in the appendage. Unfortunately, no sinoatrial node-like tissue could be found in any excised appendages. Therefore, we assumed the possibility of the existence of If channels in the appendage. Ivabradine bonded by entering and attaching to a site on the If channel pore, which prolonged the diastolic depolarization and terminated the tachycardia. However, more research is warranted to verify this assumption. Although safe and effective thus far, ivabradine may be suggested as a temporary therapy for those who fail to repeat procedures because ivabradine was unable to eliminate tachycardia. Once discontinued, tachycardia would soon recur.

**FIGURE 5:**
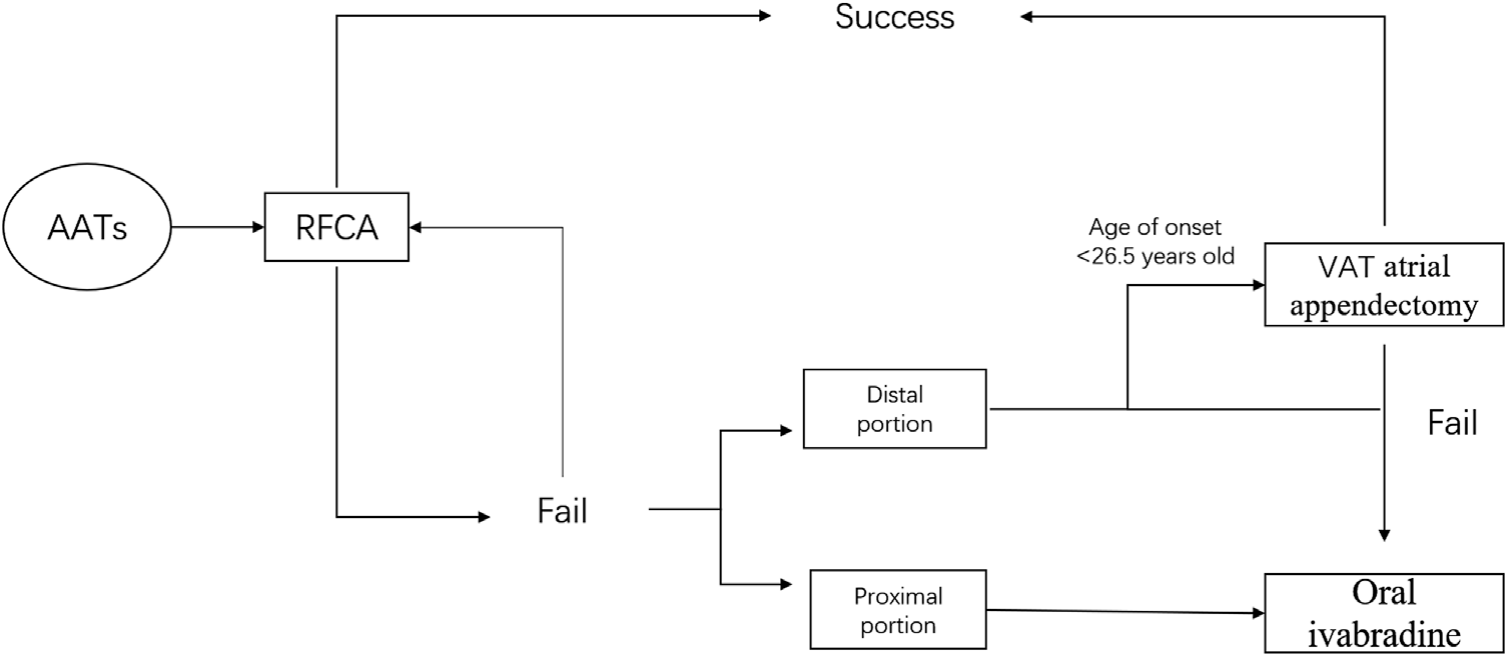
A flow chart outlining strategy for the management of AAT. AAT = atrial appendage tachycardia; RFCA = radiofrequency catheter ablation; AA = atrial appendage; VAT atrial appendectomy = video-assisted atrial appendectomy.

Based on the findings of our study, Figure 5 depicts a flow chart outlining our strategy for the management of AATs to maximize both safety and efficacy. Combined with RFCA, atrial appendectomy, and oral ivabradine treatment, a majority of AATs can be successfully managed.

### Limitations

We acknowledge that this is a single-center, retrospective study, and a combined multicenter dataset based on case series is necessary to confirm our findings. In addition, a focal cryoablation catheter was unfortunately not available for use in our institution during the study period. Had it been available, the safety and efficacy of cryoablation could be evaluated in a relatively large study cohort. Moreover, the pathogenesis of AAT is still unclear. Ivabradine was an off-label drug for treating appendage arrhythmias. There are few published reports on the use of ivabradine for treatment in pediatric and young adult patients, although AATs tend to occur in these patients. In addition, ivabradine was not inexpensive, and therefore patients could not accept such a financial burden in long-term use. Upon discontinuing ivabradine, tachycardia recurred soon. Therefore, caution was exercised for a large number of patients. In addition, whether AAT would spontaneously recover after using ivabradine should be further studied over a long-term follow-up.

## Conclusion

AAT originating from the distal AA was more refractory to RFCA. RFCA was the cornerstone of AAT catheter ablation. Video-assisted thoracoscopic atrial appendectomy was an effective strategy for those origins at the distal AA and the age of AAT onset ≤26.5 years. Ivabradine temporarily provides a promising treatment for AAT in pediatric and young adult patients.

## Data Availability

The original contributions presented in the study are included in the article/Supplementary Material; further inquiries can be directed to the corresponding authors.
